# Innovative Calcium Carbonate-Based Products to Repair Cracked Cement Mortars

**DOI:** 10.3390/ma15124044

**Published:** 2022-06-07

**Authors:** Martina Zuena, Andreja Pondelak, Enrico Garbin, Matteo Panizza, Luca Nodari, Andrijana Sever Škapin, Luka Škrlep, Gilberto Artioli, Patrizia Tomasin

**Affiliations:** 1 Institute of Condensed Matter Chemistry and Technologies for Energy, CNR, Corso Stati Uniti 4, 35127 Padova, Italy; martina.zuena@icmate.cnr.it (M.Z.); matteo.panizza@cnr.it (M.P.); luca.nodari@cnr.it (L.N.); 2 Slovenian National Building and Civil Engineering Institute, Dimičeva ulica 12, 1000 Ljubljana, Slovenia; andreja.pondelak@zag.si (A.P.); andrijana.skapin@zag.si (A.S.Š.); luka.skrlep@zag.si (L.Š.); 3 Inter-Departmental Research Centre for the Study of Cement Materials and Hydraulic Binders (CIRCe), University of Padova, Via G. Gradenigo 6, 35131 Padova, Italy; enrico.garbin@dicea.unipd.it (E.G.); gilberto.artioli@unipd.it (G.A.); 4 Faculty of Polymer Technology-FTPO, Ozare 19, 2380 Slovenj Gradec, Slovenia; 5 Department of Geoscience, University of Padova, Via G. Gradenigo 6, 35131 Padova, Italy

**Keywords:** cement mortars, 20th century architecture conservation, calcium acetoacetate, calcium alkoxide, concrete repair

## Abstract

The durability of Portland cement mortars is often affected by environmental factors, which can cause physicochemical and mechanical degradation processes. In this study, the performance of three products, calcium acetoacetate and calcium tetrahydrofurfuryloxide dissolved in two different solvents developed and tested as stone consolidants, was evaluated in terms of crack filling or sealing and consolidation. Realistic cracks were induced in quasibrittle cement mortar prisms using a custom-designed test rig. The effectiveness and the performance of the considered treatments, investigated on specimens, were evaluated by optical and scanning electron microscopy, colourimetry, water absorption rate, ultrasonic pulse velocity, and surface hardness measurements. Results revealed that, in the examined conditions, the products were more suitable as surface consolidants than as crack fillers.

## 1. Introduction

Concrete is globally the most widespread building material, and cement is used to produce more than one cubic meter of concrete per person per year [1]. Indeed, thanks to its performance when coupled with steel reinforcements, and to the capability of being cast in complex shapes, concrete ensured an unprecedented creative freedom for architects and designers, thus characterising the architecture of the 20th century since the beginning [2,3]. Furthermore, starting from modern architecture and related movements such as brutalism, which promoted aesthetic creativity via the direct exposition of construction materials, the use of béton brut [4] became an architectural feature of several paradigmatic buildings and structures, with concrete directly exposed to the external environment without paints or other protective treatments.

Initially considered to be a very durable material, in the past century, concrete has shown decay signs as a consequence of combined factors. Degradation has been mainly induced by environmental aggressiveness and internal chemical reactions, anthropogenic influences, and climatic changes, bringing to leaching, thermal and hydric expansion, shrinkage and creep, corrosion of reinforcement, and soiling [5,6]. Weathering can not only lead to aesthetic problems, but also to structural issues such as steel reinforcement corrosion [7,8,9,10].

In civil engineering, the demolition and substitution of deteriorated elements are common practice. Nonetheless, when dealing with historical and artistically valuable buildings or artefacts, other solutions (restoration, repair) should be designed and implemented. Moreover, the increased service life of concrete structures reduces the demand for new constructions and consequently raw materials, thus eventually reducing pollution, energy consumption, and CO_2_ emissions [11,12,13]. New concrete materials can include self-healing approaches, but for existing buildings, preventive repair and proper maintenance must be applied. European set of standards EN 1504 classifies two groups of possible causes of defects (degradation of concrete cover and degradation of reinforcement) and three approaches for repair methods: impregnation, sealing, and coating [14,15,16,17]. Surface treatments provide protection against external aggressive agents, while sealing is used for filling the cracks. The presence of cracks is a common feature in concrete due to its properties (porosity, composition, design): it usually does not constitute a safety problem, but depending on the width of the cracks and their diffusion (a continuous network can modify permeability), it may compromise its durability and long-term functionality by inducing loss of strength or stiffness and reinforcement corrosion [18,19,20].

Conservation treatments play a key role in the protection of construction materials, in particular in historical buildings [21,22,23,24]. Nonetheless, several products used in this field show unsatisfactory long-term performance [25,26,27]. Therefore, scientists are constantly engaged in the research of stable, efficient, compatible, and long-lasting treatments [15,18,27,28,29].

In self-healing processes that can be autogenously activated or induced with different methods [1,18,30], carbonation is the most effective in terms of crack sealing and self-healing performance. Taking this mechanism into consideration, a recent method for improving the strength of cracked brittle materials is injecting a preparation with colloidal nanoparticles of Ca(OH)_2_ able to form CaCO_3_ by reaction with the atmospheric CO_2_ [31].

Within this context, we report in the present work the application of two novel products, namely, calcium acetoacetate Ca(OAcAc)_2_ and calcium tetrahydrofurfuryloxide Ca(OTHF)_2_, to address important deterioration issues of Portland cement mortars: surface protection and the sealing of microcracks that constitute the most significant way of penetration of harmful agents (CO_2_, H_2_O, Cl^−^). The two proposed products had been developed and successfully tested as stone consolidants during European projects HEROMAT [32], and NANOMATCH [33], and they present features that can be used and optimised for cement mortar application. Both are CaCO_3_-forming compounds, hence being compatible with the inorganic composition of concrete, and both demonstrated good penetration and reconsolidation ability [34,35]. Ca(OAcAc)_2_ dissolved in water and Ca(OTHF)_2_ dissolved in a proper organic solvent can deeply penetrate porous substrates, where they transform into CaCO_3_ [36,37]. The different solubility of the used compounds renders them useful for different concrete compositions. Since Ca(OTHF)_2_ was dissolved in two different solvents, the tested products became three. Prismatic mortar samples resembling the cortical surface of a normal concrete were prepared with Portland cement. Before the application of the treatments, realistic superficial cracks were induced in the mortar prisms using a custom-designed test rig to simulate a damaged or deteriorated surface layer of concrete.

The properties of cement mortar samples were investigated before and after the cracking process and, finally, after the application of the treatments. The performance and effectiveness of the new products were evaluated in terms of efficacy and compatibility, and explored as follows: consolidation effect, penetration depth, variation in water transport properties, and changes in superficial morphological characteristics. Aesthetic performance was also evaluated because it is one of the requirements of conservation treatments for historical buildings [21].

## 2. Materials and Methods

Three products were prepared and tested. The first product, calcium acetoacetate Ca(OAcAc)_2_, is a water solution of calcium acetoacetate synthetised in the laboratory with a calcium concentration of 9.6 % [32,34]. The synthesis procedure was the following: 20 g of CaCO_3_ and 100 g of water were poured into a 1000 mL flask and stirred with a magnetic stirrer for about 10 min. Then, 61.3 g of acetondicarboxylic acid was added in small quantities. The flask was placed in a water bath with a temperature of 40 °C for 8 h. Then, 330 g of water was added to give 460 g of a 9.6% aqueous solution of calcium acetoacetate. The second, calcium tetrahydrofurfuryloxide Ca(OTHF)_2_, is a whitish-light yellow powder produced by ABCR labs (Forcarei, Spain) according to a patented method [33]. A total of 2.628 g of liquid ammonia and 339 g of metallic calcium were added to 3.01867 g of tetrahydrofuran in N_2_ atmosphere and stirred until calcium dissolution. The temperature was maintained at −65 ± 5 °C. A solution of 1.73055 g of 98% tetrahydrofurfuryl alcohol in 3.01867 g of tetrahydrofuran was added drop by drop at a temperature between −75 and −55 °C. Then, the mixture was slowly brought to ambient temperature and left overnight under vigorous stirring. Residual ammonia was removed by reduced pressure (T = 25 °C; P = 0.27 bar). An extensive characterisation of this product can be found in [35,37] and references therein. This consolidant was dissolved in either ethanol or 2-butanol to achieve the same calcium concentration as that of Ca(OAcAc)_2_. The two (analytical-grade) alcohols were purchased from Sigma-Aldrich and used as received without any further purification. Fresh nanosuspensions of Ca(OTHF)_2_ have a dispersion centered around 50 nm, with particle dimensions between 20 e 120 nm [33,35]; the aggregation of particles was reduced by sonication. Overall, the applied products were:

- Ca(OAcAc)_2_ dissolved in water (CFW);
- Ca(OTHF)_2_ dissolved in ethanol (ALK1);
- Ca(OTHF)_2_ dissolved in 2-butanol (ALK2).

Eighteen standard mortar prismatic samples (40 × 40 × 160 mm^3^) were prepared and cured according to EN 196-1:2016 [38]. One part of Portland cement CEM II/A-LL 42.5 R was mixed with 0.5 parts of tap water and 3 parts of standard quartz sand according to the reference mix design provided by EN 196-1:2016 for testing the strength of cement. The cement was selected by considering that CEM II [39] has been holding the largest market share over the last few decades in Europe compared to the other types, and limestone Portland cement is the most used among CEM II products [40,41]. Samples were cured for 28 days under water and stored in laboratory conditions at a constant temperature (20 °C ± 1 °C). 

**Figure 1:**
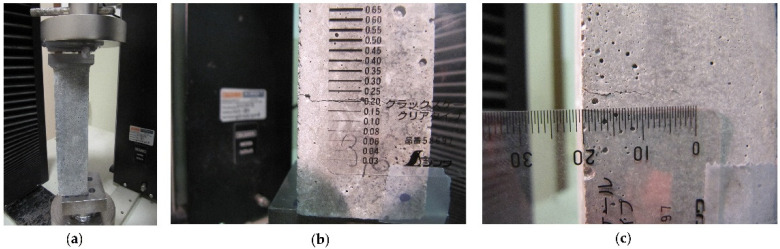
Cracking procedure: (**a**) test rig for eccentric axial compression; (**b**) width and (**c**) depth of induced cracks.

Three samples were used for the characterisation tests to measure the 28-day compressive and flexural strengths according to EN 196-1:2016 [38]. Then, 13 prisms were cracked using the custom-designed test rig shown in Figure 1a. The two remaining uncracked prisms were used as reference samples for the subsequent testing.

The cracking method and related laboratory test rig envisaged the application of an eccentric axial compression to the mortar prisms. An eccentricity of 15 mm along one of the two axes of symmetry of the 40 × 40 mm^2^ cross-section was used. This eccentricity and a proper contact with the 40 × 40 mm^2^ surfaces of the mortar prisms were produced by using a steel ball-joint and a linear hinge as bottom and top boundary conditions, respectively (Figure 1a). The linear hinge was placed parallel to one of the 40 mm sides of the cross-section and with an inward offset of 5 mm from the edge, thus producing the abovementioned eccentricity of 15 mm. The eccentric compression imposed by the boundary conditions generated combined internal plane–stress tension and compression stress field parallel to the vertical plane perpendicular to the linear hinge. Owing to the quasifragile behaviour of the cement mortar prisms [42], during loading, the internal tensile stresses reached the tensile strength of the mortar and generated the crack (Figure 1b), while the compressive stresses were still within the elastic behaviour and maintained undamaged about one-third of the 40 × 40 mm^2^ mortar cross-section. The compression load was applied in displacement control at a rate of 5 µm/s. Loading was stopped once 3 to 4 stable cracks with a mouth of about 0.2 mm (Figure 1b), a depth of 20 mm to 30 mm (Figure 1c), and spaced apart about 40 mm, had been induced in each mortar prism. 

The achievement of a crack mouth opening of about 0.2 mm was the stopping criterion for the loading procedure. After unloading, the crack mouth relaxed to about 0.1 to 0.15 mm once the eccentric axial compression had been removed from the mortar prisms, thus reproducing typical microcracks in reinforced concrete structures during their service life [43]. Hairline cracks (microcracks smaller than 0.3 mm) are usually left untreated in common practice due to the difficulty of injecting them with commercial epoxy resins [17]. Nonetheless, microcracks adversely influence the durability of reinforced concrete structures, for instance, the resistance to chloride penetration and related damage [44]. Therefore, compatible consolidants with concrete substrates and having good penetration into microcracks, and reconsolidation ability were tested in the present investigation. 

Before the application of the products, all prisms (except for the three used for the characterisation tests) were cut to obtain 40 × 40 × 40 mm^3^ cubic specimens, taking care to include at least one crack where relevant (Table S1, Supplementary Materials).

**Figure 2:**
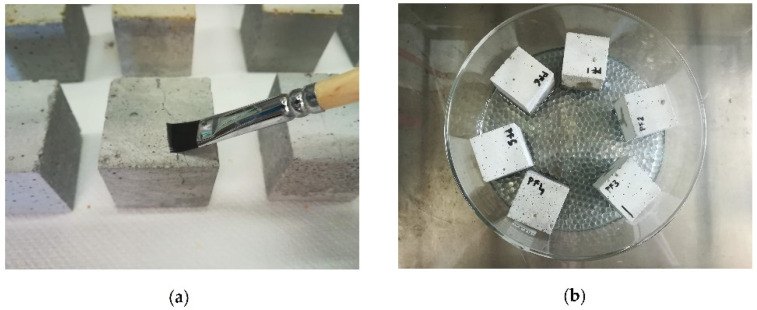
Example of application procedures of CFW: (**a**) brush till refuse, AP1; (**b**) absorption through capillarity, AP2.

Two procedures were used to apply the consolidating products: by brush till refuse (AP1), and by absorption through capillarity (AP2). In the first case, the application stopped when the surface had remained wet for more than 1 min, and it consisted of 8 applications each sample (Figure 2a). In the second case, specimens were placed over a glass sphere bed with the face to be treated left in contact with the products for 2 h (Figure 2b). For each type of test, results are expressed as the mean value of three samples.

To evaluate the amount of dry matter (kg/m^2^) retained after each application procedure and product, samples were dried and weighed before and one month after the treatment with a SalTec analytical balance.

The following measurements were carried out to characterise the performance of tested products: visual appearance via optical (OM) and scanning electron microscopy (SEM), colorimetry (COL), water absorption through capillarity (ABS), ultrasonic pulse velocity (UPV) and surface hardness (SH).

The visual appearance was investigated to detect the possible sealing of cracks and/or formation of a coating on the surface or inside the microcracks. The other techniques aimed at providing indications about aesthetic compatibility, through colorimetric measurements, and variation of physicomechanical properties (such as permeation to water, surface, and general hardness/consolidation), through water absorption tests, ultrasonic and rebound measurements.

All tests were carried out on both reference (uncracked) and cracked samples (Table S1). In the latter case, they were performed twice, before and after treatment. Multiple methods were applied to the same specimens, such as: colour measurements, water absorption through capillarity, and UPV.

**Figure 3:**
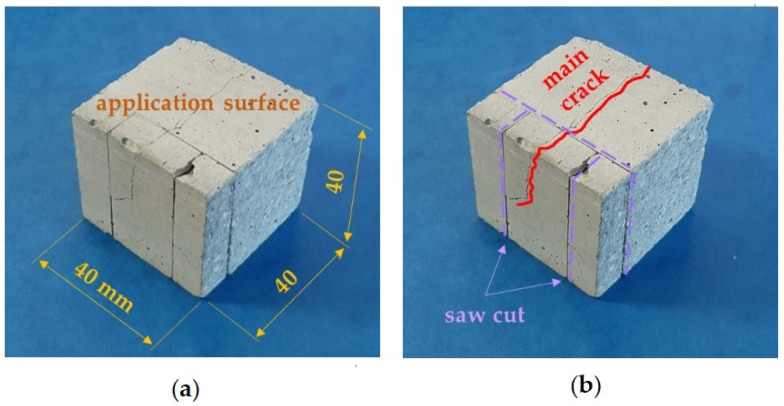
Mock-up cut to obtain smaller samples suitable for OM and SEM analysis: (**a**) application surface for the different protective treatments; and (**b**) typical location of the main crack and saw cuts.

Microscopy analyses were performed to evaluate the distribution of consolidating products. Optical microscopy was performed with an Olympus SZX12 (Olympus Corp., Tokyo, Japan) equipped with a digital camera. Four cracked samples were selected: one untreated and the remnant treated with each product: ALK1_AP2, ALK2_AP2, CFW_AP2. Application method AP2 was chosen because it guaranteed higher product penetration. Samples were analysed, without any modification, with a Scanning Electron Microscope JEOL JSM-IT500 with EDS analyser (Oxford Instruments, Tokyo, Japan) operating at low vacuum (80 Pa), at working distance of 10 mm and accelerated voltage of 15 kV, backscatter electron detector in shadow mode (BED-S). Smaller fragments (Figure 3) were also obtained through a Herbert Albert cutting saw and were analysed with an FEI Quanta 200 FEG-ESEM (FEI Czech Republic s.r.o, Brno, Czech Republic) in high vacuum conditions, after metallisation with carbon. Different magnification and detectors (BSED and EDT) were used. 

Colour measurements were performed to detect possible chromatic variations of the treated surface induced by the applied products. A spectrophotometer Konica Minolta CM-700d, (Konica Minolta Corp., Tokyo, Japan) under Illuminant D65 at a 10° of observation was adopted according to NORMAL 43/93 [45]. For each treatment, three samples were analysed, and four points for each sample were considered. Results are reported in the CIE L*a*b* colour space, and the total colour difference ∆E* was calculated as follows (Equation (1)):

(1) $$\Delta E^{*}=\sqrt{\Delta L^{*2}+\Delta a^{*2}+\Delta b^{*2}}$$

where L* is the lightness; a* and b* are the colorimetric coordinates for red/green and yellow/blue parameters, respectively (a* < 0 green, a* > 0 red, b* < 0 blue, and b* > 0 yellow); Δ is the difference between the mean values of the treated and untreated surfaces. Data were elaborated with the Spectramagic software.

Water absorption through capillarity was performed under the procedure provided by standard UNI 10859:2000 [46]. All the surfaces of the sample were covered with aluminium tape except the treated one, which was then put in contact with water. Samples were weighed at regular time intervals until a steady-state had been reached. A comparison before and after treatment was obtained by calculating the capillary water absorption coefficient (CWAC), which is the angular coefficient of the first part of the capillary absorption curve. For each treatment, results are reported as the mean of three samples.

Ultrasonic pulse velocity (UPV) is one of the most used undestructive techniques to evaluate the status of concrete, to detect internal defects, and to estimate the crack depth and compressive strength [47,48,49]. It can be correlated to stiffness (e.g., modulus of elasticity), compressive strength, porosity, and permeability. Indeed, an increase in wave velocity generally indicates an improvement in material cohesion. In our case, measurements before and after the application of treatments can indicate a crack filling and/or a consolidation action. A Pundit transmitter (London, UK) with a frequency of 54 kHz was used. Measurements were taken with the direct method by placing transducers on the opposite surfaces perpendicularly to the treated one. Results are expressed as the mean value of three measurements.

Surface hardness measurements were performed through a portable tester Equotip 3 (Proceq, Zurich, Switzerland), which is based on the Leeb dynamic rebound test method [34]. Probe D, which has an impact energy of 11 N mm, was used. The hardness value is expressed as the Leeb hardness (HLD), i.e., the ratio of rebound velocity to impact velocity multiplied by 1000 [50]. Results are given as the average of 10 measurements obtained for each sample (3 samples for treatment).

A detailed list of all the abbreviations used in this paper is reported in Table S2 (Supplementary Material).

## 3. Results and Discussion

Characterisation tests on the cement mortar prisms delivered an average compressive strength of 42.1 MPa (CoV 0.8%) and an average flexural strength of 6.3 MPa (CoV 2.0%). Measured strengths were in line with the type of tests and used cement, thus confirming the proper preparation and curing of mortar specimens.

### 3.1. Retained Dry Matter of Consolidants

Table 1 reports the average amount of dry matter retained by cubic samples treated with the three products, each of them applied with both methods. As expected, method AP2 lead to a greater amount of retained material compared to AP1, about 15–20% for products ALK1 and ALK2 and more than 140% for CFW. ALK1 and ALK2 showed similar results on equal methods, while the amount of retained material for CFW, compared to the former, was slightly higher (6%) in the case of AP1, and remarkably greater (more than 110%) in the case of AP2.

The lower quantity of alkoxide-based solutions could have been due to some ageing of the applied product, as already observed [35]. The product received from ABCR was not whitish as usual, but light yellow, an indication of incomplete purification, which can induce the faster ageing of the product. 

### 3.2. Visual Appearance

**Figure 4:**
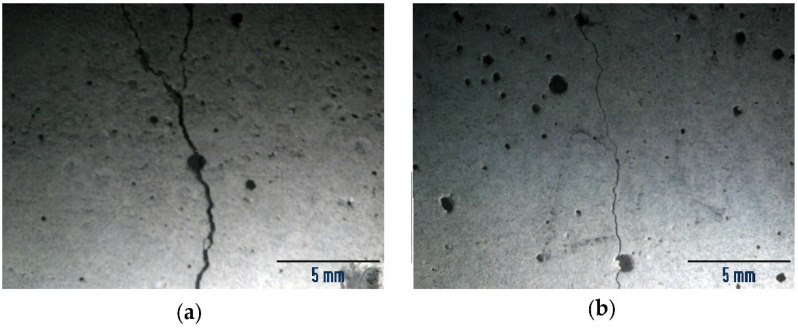
Stereomicroscopy images (7×) of samples (**a**) untreated and (**b**) treated with ALK2_AP1: a reduction in crack width is visible.

Observations of the application face of samples through optical microscopy on representative samples of all the applied products (one for each product and treatment and 2 not treated as references, 8 samples in total) generally seemed to reveal a reduction in the width of the cracks after the introduction of the consolidants (Figure 4a,b, referring to ALK2_AP1). This was a general trend, although measurements were not performed on the same samples before and after application.

**Figure 5:**
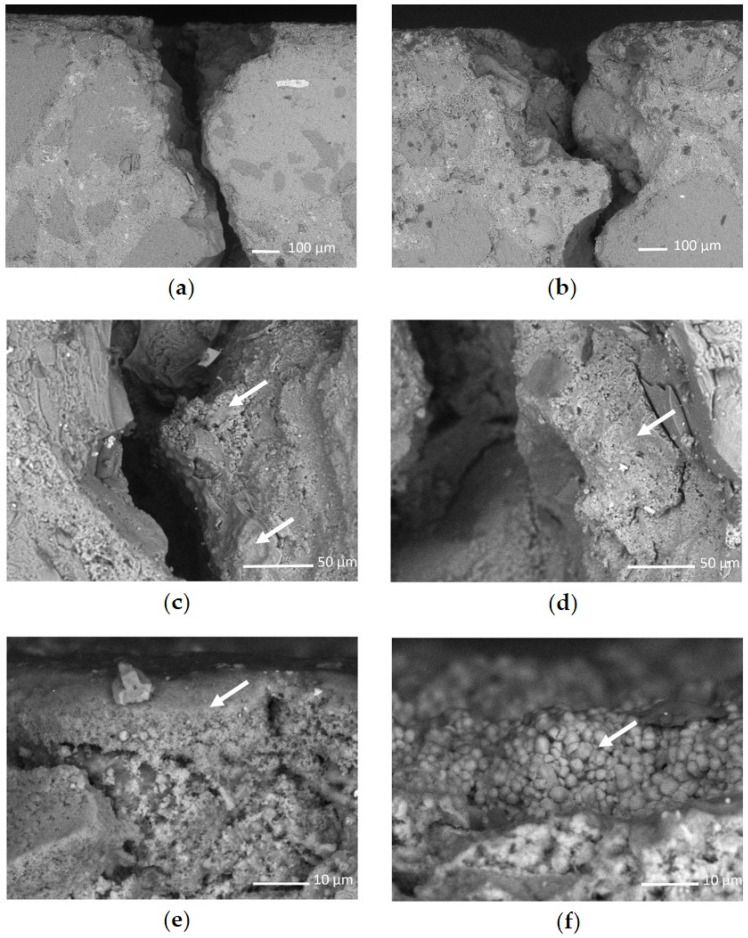
SEM images of samples treated with (**a**,**c**,**e**) ALK1 and (**b**,**d**,**f**) CFW, applied via absorption through capillarity (AP2), at different magnifications ((**a**,**b**): 100×; (**c**,**d**): 500×; (**e**,**f**): 2000×). (**a**,**b**) At low magnification, it is evident that the crack was not filled with consolidants; (**c**,**d**) at higher magnifications, the consolidant material was deposited on the crack walls. At even higher magnifications, the morphology of different consolidants was clearly seen to be (**e**) needlelike and (**f**) spherical particle formation.

Four selected cubic mock-ups (one untreated and one treated by absorption through capillarity for each product: ALK1_AP2, ALK2_AP2, CFW_AP2), observed in advance with optical microscopy, were then observed with the scanning electron microscope (JEOL instrumentation) to evaluate a possible crack-filling effect. SEM images of samples treated with ALK1 and CFW by immersion (AP2) are shown in Figure 5. At low magnifications, consolidants apparently did not exhibit a crack-filling effect (Figure 5a,b). Higher magnifications of cracks show that consolidants were deposited on the crack walls, as indicated by the arrows in Figure 5c,d: on the surface of samples, the formation of needlelike (Figure 5e) and spherical (Figure 5f) particles can be seen. Needlelike particles are characteristic for alkoxide transformation [51], while spherical particles are characteristic for vaterite formation, typically occurring in CFW transformation [52]. Formed vaterite particles are approximately 1 to 3 µm in size, which is in accordance with previous studies [52].

It is interesting to compare these results with those obtained from the use of the other SEM instrumentation (FEI Quanta 200), where the preparation of samples required a rather aggressive cutting in presence of water. In one case (ALK1_AP2), the deposit of the treatment inside the cracks was evident, but with an inhomogeneous distribution. For CFW_AP2 and ALK2_AP2, the deposit was scarcely visible in the cracks; however, CFW seemed to have a more homogeneous distribution. The procedure used to cut the samples might have partially removed the coating, as also observed by Roig-Flores and Serna [53].

### 3.3. Colorimetry

**Figure 6:**
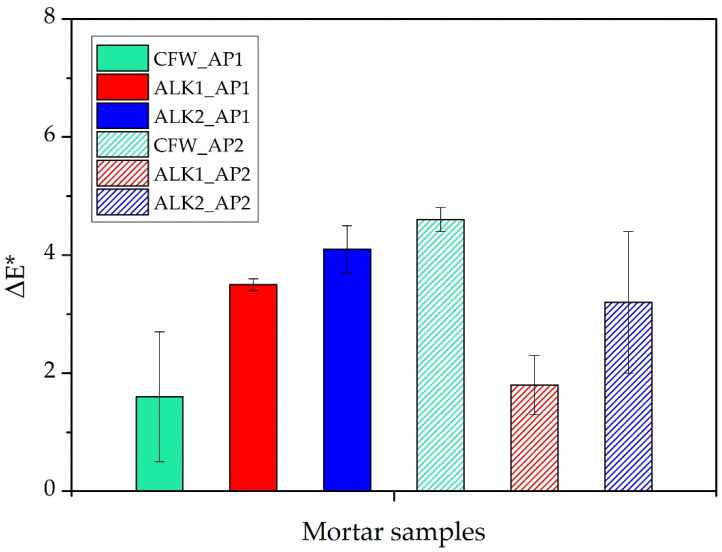
Chromatic variation after the application of all products with both procedures. CFW: Ca(OAcAc)_2_; ALK1: Ca(OTHF)_2_ in ethanol; ALK2: Ca(OTHF)_2_ in 2-butanol; AP1: brush till refuse; AP2: absorption through capillarity.

The surface colour variation caused by treatments was, in all cases, below the acceptable limit ΔE* < 5 (according to García and Malaga [54]), which is hardly appreciable by visual estimation, as shown in Figure 6. This datum suggests their aesthetical compatibility with the treated support. Indeed, this is an important parameter when proposing a treatment for historical buildings and cultural heritage objects, where visual integrity must be preserved [21,55].

### 3.4. Water Absorption

As water plays an essential role in many deterioration mechanisms of concrete, high resistance to its penetration is desirable for surface treatments [53]. Water absorption, which is closely related to concrete pore structure, might provide indirect information about the effectiveness of treatments in this regard, reflecting possible changes of porosity before and after consolidation.

**Figure 7:**
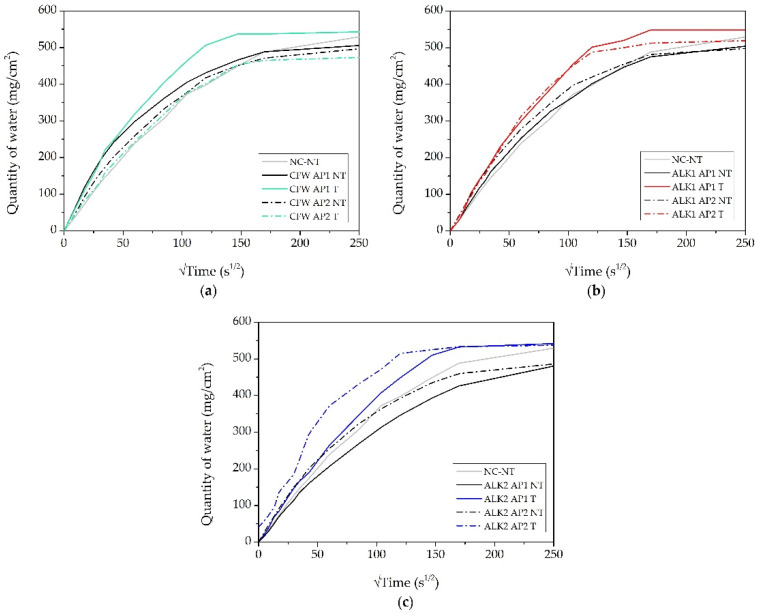
Quantity of water absorbed by capillarity–comparison among uncracked–untreated samples (NC–NT), cracked–untreated samples (CFW_AP1 NT, CFW_AP2 NT, ALK1_AP1 NT, ALK1_AP2 NT, ALK2_AP1 NT, ALK2_AP2 NT) and cracked–treated samples (CFW_AP1 T, CFW_AP2 T, ALK1_AP1 T, ALK1_AP2 T, ALK2_AP1 T, ALK2_AP2 T): (**a**) focus on CFW (**b**) focus on ALK1 and (**c**) focus on ALK2. CFW: Ca(OacAc)_2_; ALK1: Ca(OTHF)_2_ in ethanol; ALK2: Ca(OTHF)_2_ in 2-butanol; AP1: brush till refuse (continuous lines); AP2: absorption through capillarity (dashed lines).

Water absorption tests were carried out for all the samples, and the results are reported in Figure 7 and Table 2. After the application of the products, water absorption was generally similar to that of the untreated mortars. The water absorption of CFW_AP2 decreased during the first 8 h when compared to the cracked–untreated samples (NT) and to the other treatments with both application procedures, which could have been related to the maximal material uptake into the fracture (Table 1).

In general, products did not affect the water absorption, although a small reduction in CWAC was considered to be related to the efficacy of a consolidation treatment. However, untreated samples start with a low CWAC; therefore, it is difficult to have consistent variation in this parameter. Furthermore, a variation in CWAC of less than 10% is generally suggested [56] to assess the compatibility of a consolidation product. Regarding ALK1_AP1, which showed an unusual increase in CWAC, the standard deviation was significant, probably due to the different morphology of the cracks and inhomogeneity among samples. However, more in-depth investigations might suggest alternative hypotheses.

### 3.5. UPV Measurements and Consolidation Effect

UPV measurements were performed on uncracked and cracked specimens, the latter both before and after the application of treatments. Results (Table 3) show that the recorded values of ultrasound velocity after the application of consolidants increased in all cases and were slightly greater when compared to the reference uncracked samples.

Therefore, a certain consolidation effect was observed for all treatments with both application procedures. The major differences before and after treatment were with application method AP2, except for CFW. ALK1 and ALK2 had the best performance in percentage with the application by capillarity, although their retained quantity of product was the lowest. The higher UPV values after treatment were probably due to the deposition of the consolidant on the surfaces of the cracks, as can be seen from SEM images in Figure 5c,d.

### 3.6. Surface Hardness

**Figure 8:**
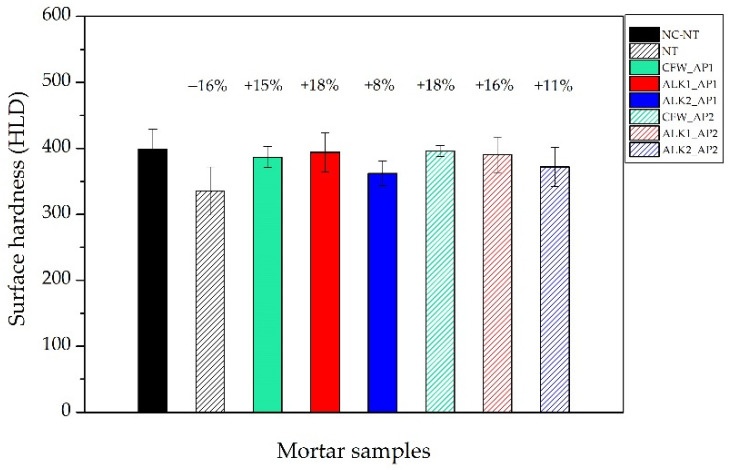
Comparison of surface hardness among: uncracked–untreated samples (NC–NT), cracked–untreated samples (NT) and cracked–treated samples. CFW: Ca(OAcAc)_2_; ALK1: Ca(OTHF)_2_ in ethanol; ALK2: Ca(OTHF)_2_ in 2-butanol; AP1: brush till refuse; AP2: absorption through capillarity.

Rebound data of reference samples and specimens treated with all the products and their percentages of variation (%) are presented in Figure 8. The percentage of variation (%) of cracked–untreated samples (NT, −16%) was calculated concerning to untreated and uncracked samples (NC–NT); the results referring to treated samples are expressed with respect to the cracked–untreated samples. In this case, tests were not performed on the same specimens before and after the treatment, but the reference was provided by a separate set of samples. Figure 8 reports in more detail the relative variation in surface hardness, referring to either uncracked–untreated results (NC–NT) for cracked–untreated samples (NT) or to the latter for treated ones.

The presence of cracks on untreated samples (NT) led to a decrease of 16% with respect to intact NC–NT, while treatments where able to increase the surface hardness to similar values to those of uncracked–untreated samples, with the exception of ALK2, which showed an improvement of between 50% and 70% compared to the average performance of the other treatments. This may provide weak protection of damaged or altered surfaces to abrasion.

## 4. Conclusions

This paper reported a preliminary assessment of the possible use of calcium acetoacetate (in water solution) and calcium tetrahydrofurfuryloxide (dissolved in either ethanol or 2-butanol) as products for the repair of deteriorated cement mortars.

The various analyses performed on reference and treated samples provided a first interesting insight on the use of these treatments for cement mortars.

First, the retained quantity of applied products did not show straightforward correlation to their measured results. Ultrasonic and surface hardness measurements showed a certain consolidation effect promoted by all treatments regardless of agent or application method. Furthermore, no significant chromatic variation of the surface was observed, suggesting their suitability for applications to cultural heritage artefacts. Treatments, however, did not affect the CWAC, which remained constant in almost all cases. In addition, SEM observations revealed that the applied products formed particles that had been deposited on the crack walls.

Specific effects can be noted. Alkoxide in ethanol (ALK1) determined a relevant increase in surface hardness when applied by brush, while it did not achieve remarkable performance as consolidant. This was likely due to the application method, i.e., by brush, that did not allow for sufficient penetration of the product in the small and large fractures. Conversely, both alkoxides applied by capillarity enhanced UP velocity and surface hardness, suggesting a more effective and deep penetration into the substrate. Calcium acetoacetate showed this trend regardless of application method.

Generally speaking, measured differences between reference and treated samples were rather limited in all cases because the recorded values, also for the cracked samples, were typical of a healthy cement mortar (see Table 1 in Karaiskos et al. [49]). Harsher weathering conditions applied to specimens might more clearly highlight the possible consolidation effects. In addition, the application of UPV probes on the lateral faces of specimens (orthogonal with respect to cracks) might provide better evaluation of penetration and crack filling effects of treatments [47,48,49].

Nonetheless, these products showed potential as compatible surface consolidants for concrete repair, provided that further research investigates the use of more concentrated solutions and the effect of multiple applications injected in the cracks, and the effects on aged and weathered surfaces. 

## Figures and Tables

**Table 1:** Quantity of dry matter retained after one month for all products and application procedures.

Samples ^1^	Dry Matter (kg/m^2^)
CFW_AP1	0.57 ± 0.08
ALK1_AP1	0.54 ± 0.08
ALK2_AP1	0.54 ± 0.07
CFW_AP2	1.37 ± 0.15
ALK1_AP2	0.63 ± 0.07
ALK2_AP2	0.65 ± 0.07

^1^ CFW: Ca(OAcAc)_2_; ALK1: Ca(OTHF)_2_ in ethanol; ALK2: Ca(OTHF)_2_ in 2-butanol; AP1: brush till refuse; AP2: absorption through capillarity.

**Table 2:** Comparison of capillary water absorption coefficient (CWAC) before and after treatment.

Samples ^1^	CWAC	
	Before Treatment	After Treatment
CFW_AP1	5.9 ± 0.6	5.9 ± 0.1
ALK1_AP1	4.6 ± 0.3	5.2 ± 0.5
ALK2_AP1	3.8 ± 0.2	3.8 ± 0.3
CFW_AP2	5.1 ± 0.4	4.2 ± 0.2
ALK1_AP2	5.3 ± 0.2	5.2 ± 0.3
ALK2_AP2	4.8 ± 0.1	4.9 ± 0.3

^1^ CWAC: capillary water absorption coefficient. CFW: Ca(OacAc)_2_; ALK1: Ca(OTHF)_2_ in ethanol; ALK2: Ca(OTHF)_2_ in 2-butanol; AP1: brush till refuse; AP2: absorption through capillarity.

**Table 3:** UPV values for uncracked–untreated and for cracked samples, before and after treatment.

Samples ^1^	UPV (m/s)		%
	Before Treatment	After Treatment	
NC-NT	4134.0 ± 28.5	–	
CFW_AP1	4080.4 ± 56.4	4257.7 ± 33.1	+4.3
ALK1_AP1	4171.2 ± 57.3	4243.9 ± 6.3	+1.7
ALK2_AP1	4201.7 ± 96.3	4274.9 ± 54.4	+1.7
CFW_AP2	4172.6 ± 53.1	4307.2 ± 48.9	+3.2
ALK1_AP2	4120.7 ± 46.6	4307.9 ± 21.9	+4.5
ALK2_AP2	4085.6 ± 43.8	4289.3 ± 41.4	+5.0

^1^ NC–NT: uncracked–untreated samples; CFW: Ca(OAcAc)_2_; ALK1: Ca(OTHF)_2_ in ethanol; ALK2: Ca(OTHF)_2_ in 2-butanol; AP1: brush till refuse; AP2: absorption through capillarity.

## Data Availability

Not applicable.

## Supplementary Materials

The following supporting information can be downloaded at: https://www.mdpi.com/article/10.3390/ma15124044/s1, Table S1: list of the analysed samples. Table S2: legend of the used abbreviation concerning the used products, type of applications, samples and measurements.
