# Supplementary material for: Innovative Calcium Carbonate-Based Products to Repair Cracked Cement Mortars
Source: Materials (Basel). 2022 Jun 7;15(12):4044. doi: 10.3390/ma15124044 (PMC9229066; doi:10.3390/ma15124044)
Supplement: Supplementary file 1 [file materials-15-04044-s001.zip › materials-1745820-supplementary.pdf]

Supplementary materials

Table S1: list of the analysed samples.

**Sample**	**Crack procedure**	**Treatment**	**Type of application**	**Proof**
**ST1**	Not cracked	Untreated	-	COL/UPV/ABS/SH
**ST2**	Not cracked	Untreated	-	COL/UPV/ABS/SH
**ST3**	Not cracked	Untreated	-	COL/UPV/ABS/SH
**ST7**	Cracked	Untreated	-	SH/OM
**ST8**	Cracked	Untreated	-	SH/OM/SEM
**ST9**	Cracked	Untreated	-	SH
**PA1**	Cracked	ALK1	AP1	COL/ABS/UPV
**PA2**	Cracked	ALK1	AP1	COL/ABS/UPV/OM
**PA3**	Cracked	ALK1	AP1	COL/ABS/UPV
**PB1**	Cracked	ALK2	AP1	COL/ABS/UPV/OM
**PB2**	Cracked	ALK2	AP1	COL/ABS/UPV
**PB3**	Cracked	ALK2	AP1	COL/ABS/UPV/OM
**PC1**	Cracked	CFW	AP1	COL/ABS/UPV
**PC2**	Cracked	CFW	AP1	COL/ABS/UPV
**PC3**	Cracked	CFW	AP1	COL/ABS/UPV
**PD1**	Cracked	ALK1	AP2	COL/ABS/UPV
**PD2**	Cracked	ALK1	AP2	COL/ABS/UPV
**PD3**	Cracked	ALK1	AP2	COL/ABS/UPV/OM/SEM
**PE1**	Cracked	ALK2	AP2	COL/ABS/UPV
**PE2**	Cracked	ALK2	AP2	COL/ABS/UPV
**PE3**	Cracked	ALK2	AP2	COL/ABS/UPV/OM/SEM
**PF1**	Cracked	CFW	AP2	COL/ABS/UPV/OM/SEM
**PF2**	Cracked	CFW	AP2	COL/ABS/UPV
**PF3**	Cracked	CFW	AP2	COL/ABS/UPV
**PA4**	Cracked	ALK1	AP1	SH
**PA5**	Cracked	ALK1	AP1	SH
**PA6**	Cracked	ALK1	AP1	SH
**PB4**	Cracked	ALK2	AP1	SH
**PB5**	Cracked	ALK2	AP1	SH
**PB6**	Cracked	ALK2	AP1	SH
**PC4**	Cracked	CFW	AP1	SH
**PC5**	Cracked	CFW	AP1	SH
**PC6**	Cracked	CFW	AP1	SH
**PD4**	Cracked	ALK1	AP2	SH
**PD5**	Cracked	ALK1	AP2	SH
**PD6**	Cracked	ALK1	AP2	SH
**PE4**	Cracked	ALK2	AP2	SH
**PE5**	Cracked	ALK2	AP2	SH

**PE6**	Cracked	ALK2	AP2	SH
**PF4**	Cracked	CFW	AP2	SH
**PF5**	Cracked	CFW	AP2	SH
**PF6**	Cracked	CFW	AP2	SH

ALK1: $Ca(OTHF)_2$ in ethanol; ALK2: $Ca(OTHF)_2$ in 2-buthanol; CFW: $Ca(OAcAc)_2$; AP1: brush till refuse; AP2: absorption through capillarity; ABS: absorption though capillarity; COL: colorimetric measurement; OM: Optical microscopy; SEM: scanning electron microscopy; SH: surface hardness; UPV: ultrasonic pulse velocity.

Table S2: legend of the used abbreviation concerning the used products, type of applications, samples and measurements.

Abbreviation	Type	Meaning
**CFW**	Product	Calcium acetoacetate $Ca(OAcAc)_2$
**ALK1**	Product	Calcium tetrahydrofurfuryloxide $Ca(OTHF)_2$ dissolved in ethanol
**ALK2**	Product	Calcium tetrahydrofurfuryloxide $Ca(OTHF)_2$ dissolved in 2-butanol
**AP1**	Application	Application by brush till refuse
**AP2**	Application	Application by absorption through capillarity
**NT-NT**	Sample	Uncracked-untreated samples
**NT**	Sample	Cracked-untreated samples
**OM**	Measurement	Optical microscopy
**SEM**	Measurement	Scanning electron microscopy
**COL**	Measurement	Colorimetry
**ABS**	Measurement	Water absorption through capillarity
**UPV**	Measurement	Ultrasonic pulse velocity
**SH**	Measurement	Surface hardness
